# Linguistic Processing and Classification of Semi Structured Bibliographic Data on Complementary Medicine

**DOI:** 10.4137/cin.s1182

**Published:** 2009-07-06

**Authors:** Thomas Ostermann, Christa K. Raak, Peter F. Matthiessen, Arndt Büssing, Hartmut Zillmann

**Affiliations:** 1 Chair of Medical Theory and Complementary Medicine, Faculty of Medicine, University of Witten/Herdecke, Gerhard-Kienle-Weg 4, 58313 Herdecke, Germany; 2 Specialist Library for Complementary Medicine, University of Witten/Herdecke, Gerhard-Kienle-Weg 4, 58313 Herdecke, Germany; 3 IDM—Intelligent Data Management, Osnabrueck, Germany

**Keywords:** cancer, complementary medicine, database, semantic web, literature

## Abstract

Complementary and alternative therapies and medicines (CAM) such as acupuncture or mistletoe treatment are much asked for by cancer patients. With a growing interest in such therapies, physicians need a simple tool with which to get an overview of the scientific publications on CAM, particularly those that are not listed in common bibliographic databases like MEDLINE. CAMbase is an XML-based bibliographical database on CAM which serves to address this need. A custom front end search engine performs semantic analysis of textual input enabling users to quickly find information relevant to the search queries. This article describes the technical background and the architecture behind CAMbase, a free online database on CAM (www.cambase.de). We give examples on its use, describe the underlying algorithms and present recent statistics for search terms related to complementary therapies in oncology.

## Introduction

In 1999, Zollmann and Vickers defined complementary and alternative medicine (CAM) as “a group of therapeutic and diagnostic disciplines that exist largely outside the institutions where conventional health care is taught and provided”).^1^ In the last decade however, complementary medicine has not only succeeded in establishing itself in the academic context, with an increasing number of institutions at European universities working on CAM, it has also expanded its activities in all fields of research.^2^

The growing academic development of CAM accompanies the increasing demand for complementary therapies in the public domain. All surveys of this field show that, depending on the country, between 20% and 65% of people use CAM,^3^ and that patients often request to be updated on CAM options. Although the family physician is in most cases regarded as the first choice for such inquires, a growing number of patients also use the internet as a resource for CAM information. In one survey, the Pew Internet and American Life Project found that 48% of health seekers have looked for information about CAM on the internet.^4^

In particular, there is an increasing demand for information on CAM among cancer patients.^5^ Patients are actively seeking treatments and are thus aiming at utilizing coping strategies which might be helpful to extend survival time, gain a better quality of life or to relieve pain.^6^,^7^ While many patients have discovered the World Wide Web as a resource for information on CAM,^8^ modest empirical research has been conducted into the kind of sources of CAM information and search tools used by cancer patients. Some observations suggest that cancer patients increasingly search professional sources of clinical evidence in the internet, such as patient guidelines or databases.^9^ Our own experience with CAMbase, a bibliographic database on CAM, revealed a strong interest from cancer patients in evidence based information on complementary therapies.^10^

**Figure 1:**
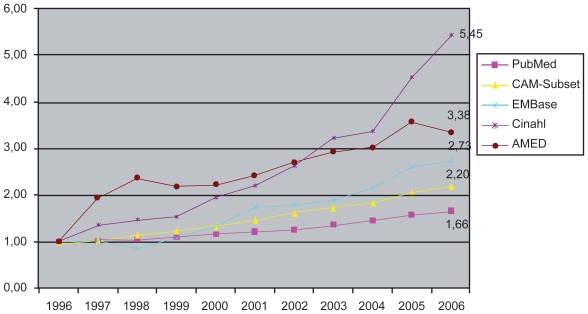
Relative growth of bibliographical datasets in various online-sources of information on the search query term “cancer” from 1996 to 2006. Absolute number of hits for “cancer” in 1996: Pubmed: 56070; CAM-Subset of Pubmed: 2611; EMBase: 290; Cinahl: 451; AMED: 208.

The increasing interest of cancer patients in the internet and the diversity of information resources has led to difficulties in identifying, filtering and implementing new and appropriate medical knowledge. Figure 1 illustrates the growth of information in various bibliographic databases using the example of the search term “cancer”. Between 1996–2006, there was an increase in information in resources such as PubMed of 66% (CAM-Subset: 120%), Allied and Complementary Medicine Database (AMED) 238% and “Cumulative Index to Nursing and Allied Health (CINAHL) 454%.

The expansion of the internet in the last ten years has made information retrieval challenging not only for patients but also for health care providers and physicians who wish to separate “important/meaningful” from “unimportant/meaningless” information.^11^ Thus, with the growing interest in CAM, there is an urgent need by users of online databases for tools to assist in reviewing the scientific information on CAM in cancer.

Despite the development of CAM research and application in recent decades, a large proportion of the publications reporting on CAM cannot be easily found in digital bibliographic databases like MEDLINE. This is mostly due to two problems:

1. A broadly accepted thesaurus for CAM in total does not exist: With respect to heterogeneity, CAM has not developed a strong tradition for a controlled vocabulary to classify CAM literature^12^ and despite some promising efforts,^13^ this is still an unsolved problem. In addition, the conventional MESH-Keywords of MEDLINE do not adequately map the contents of CAM-Literature.
2. Despite the existence of semi-structured bibliographic data on CAM in electronic databases, a researcher might use misleading or ambiguous keywords in his search strategy and hence cannot find the relevant data. This problem is also known as the so called “vocabulary problem”.^14^

While the first problem is a result of the diverse composition of CAM itself, the second also arises in other biomedical contexts and is a problem on the client’s side.

In our work, we have sought to provide search options suitable for (i) experienced users who want to employ a specific search strategy, e.g. for systematic reviews, as well as for (ii) casual users who want to inform themselves on a topic which at that moment of query issue might not be paraphrased very precisely.^15^

This article describes the linguistic processing and classification of semi-structured bibliographical data on complementary and alternative medicine in CAMbase, a free bibliographic database on CAM realized with the semantic web standard XML and accessible online at the URL www.cambase.de.

## Technical Realization

In situations where there is no concise repertoire of controlled vocabulary, there is a necessity to build tools which guide the user through the bibliographical landscape. Based on the inverted file structure and the implementation of XML together with the linguistic algorithms, we are able to offer such a search-tool of CAM-landscapes, which are created for every single search query based on elementary bibliographic data (i.e. keywords or authors).

The basic idea behind this approach is to guide the user through the mass of resulting datasets, which in the case of the search query ‘cancer’ in conventional databases like MEDLINE might lead to a large volume of results as can be seen in Figure 1.”

In the following section we focus on the specific topics of processing and classification of semi-structured bibliographical data.

## Search Procedures and Strategies

Search strategies in medical research, e.g. for systematic reviews, are nowadays targeted towards identifying relevant articles in bibliographical databases such as MEDLINE and are commonplace.^16^ Users are primarily scientists or specially trained library professionals, who are consulted by domain experts. On the other hand, an increasing number of physicians are embracing the internet to gather information about the latest guidelines or to deepen their education in web-based medical training and seminars.^17^ When the structure of CAMbase was initially planned, we intended to develop search options which suit both of these user-groups. Therefore, in addition to conventional search options (Author, title, keywords, publication year, etc.), we implemented a natural language interface with linguistic algorithms designed to facilitate enhanced query capture before issuing to the search process.

Apart from conventional techniques of natural language processing (NLP) like stemming, text segmentation and the analysis of punctuation and normalisation (see Brants^18^ for a review of common methods of NLP), we have developed special features, which are quite unique with respect to their decomposition of search phrases into their linguistic and grammatical entities. In the following we illustrate these features using two examples:

### Example 1: Modification and restriction of a subject, explicitly formulated by the user

**Figure 2:**
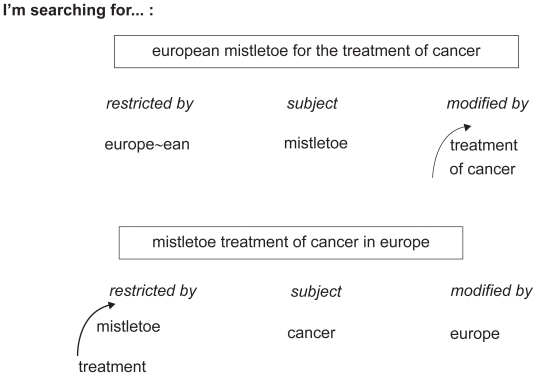
Linguistic processing of natural language queries: Subject Search of “European mistletoe for the treatment of cancer” leads to different results than “mistletoe treatment of cancer in Europe.”

In the search term “*European mistletoe for the treatment of cancer*”, the phrase “mistletoe” is the subject modified by “treatment of cancer” and restricted by “Europ~ean”, whereas in the search term “*mistletoe treatment of cancer in Europe*”, the term “cancer” is the central subject modified by “Europe” and restricted by “mistletoe treatment” (Fig. 2). Thus, even though the textual phrase consists of the same words, the ranking of the search results will be different^19^–^21^ because of the grammatical relation between the words. Note that most of these language processing features are quite unique for German.

As can be seen in this example, linguistic procedures like automated morphological reduction and decomposition algorithms also help in the processing of search queries: the search query “european” will also lead to hits on “europe” and the German search query “Krebstherapie” (cancer therapy) also finds “Therapie des Krebses” (therapy of cancer) and vice versa. Table 1 gives an overview of the implemented NLP-techniques.

## Ranking Algorithm

Based on a mathematical analysis of how the search phrase is conceptually represented in the dataset, a numerical distance value between 0 (=no relevance) to 100 (identical) is computed, which helps the user to decide which documents fit to his/her search query, i.e. datasets with a higher ranking will be placed in a higher position than those with a lower ranking. The underlying algorithm can be described as follows:

Let *C* = {*d|d =* 1 *… D*} denote the *corpus* containing a total of D datasets *d* and let *W_d_* = {*w_i_^d^*|*w_i_^d^* ∈ *d; i* = 1 . . .*n*} denote the set of all words *w_i_^d^* of the dataset *d.* The inclusion of words depends on the given record structure and the decision of the database owner. In our case we included all words of bibliographical fields (i.e. title, keywords, abstract) and excluded content notes such as page numbers and stopwords such as “the”,“an” or “and”, which were filtered out prior to the processing. Based on the *w_i_^d^* an address vector *w⃗_i_^d^* = (*w_i_^d^*, *a*_1_, *a*_2_, . . ., *a_k_*) is defined where *a*_1_, *a*_2_, *…*, *a_k_* denote additional coded information about syntactical and morphological attributes of the word *w_i_^d^* (See Table 1 for an overview of these additional informations). Then *W̃_d_* = {*w⃗_j_^d^*|; *i* = 1 . . . *ñ*} is called the *index* of the dataset *d* and *W̃* = ∪ *W̃_d_* is called the *index* of the whole database. Finally the terms *S_j_* of the search query *S* are defined by *S*={*s_j_*|*s_j_*; *j* = 1 . . .*m*} Again, according to the construction of the *w⃗_i_^d^* a search vector *s⃗_j_* = (*s_j_*, *a*_1_, *a*_2_, . . ., *a_k_*) is defined. The search query *S* and all algorithms are processed on the index *W̃*.

Independent of the location of the terms *s_j_* ∈ *W̃_d_*∀*j* = 1 . . . *m̃, m̃* ≤ *m* we define a parameter 0 < *Q_m̃_*≤100 for these datasets. Note that *s_j_* ∈ *W̃_d_* also is fulfilled if *S_j_* is ‘part’ of a word *w_i_^d^* (i.e. the German term “*Therap~ie*” in “*Misteltherap~ie*” or the English term “*therap~y*” *in* “*therap~eutic*”). In the case of a normally distributed heterogeneity of *C*, *Q_m_* like in our case this value is set to 50 and *Q_m̃_* are defined accordingly as *Q_m_*· *m̃/m.*

Based on *Q_m̃_* the quality function $Q_{d}(S)=Q_{\tilde{m}}+\sum_{j=1}^{m}\sum_{i=1}^{n}q_{i}({\vec{w}}_{i}^{d},{\vec{s}}_{j})\leq 100$ is calculated, where *q_i_*(*w⃗_i_^d^* *s⃗_j_*) denote additional quality criteria functions (i.e. correct flexion, correct position and order of the words, containment in a compound word, irregular plurals) which are given by the *a*_1_, *a*_2_, . . ., *a_k_*. Depending on their weightings, the *q_i_*(*w⃗_i_^d^* *s⃗_j_*) add a significant amount to the value of *Q_m̃_* Finally the quality threshold *Q_t_* = *Q_m_*−*δ_m_*;*δ*> 0 is defined as the lower bound for the quality of a dataset. In other words, every dataset *d* with a value *Q_d_*(*S*) ≥ *Q_t_* is presented as a result of the search query *S.* Note that all datasets with *s_j_* ∈ *W̃_d_*∀*j* = 1 . . . *m* by definition are above the threshold of *Q_t_*. If wanted, both the threshold value and the sensitivity of the *q_i_*(*w⃗_i_^d^* *s⃗_j_*) may be adjusted by the user of the database. In the case of CAMbase, *Q_t_* is set to the value of 45.

**Figure 3:**
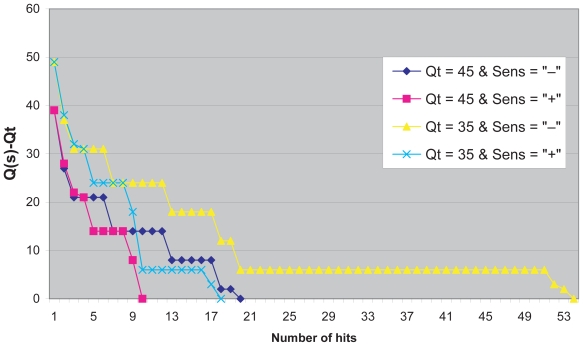
Behaviour of the search algorithms for the threshold values *Q_t_* = 35 and *Q_t_* = 45 and high (+) versus low (−) sensitivity. The quality index *Q*(*s*) −*Q_t_* is plotted against the number of hits and produces different survival curves for the datasets searched with the query “adverse effects of mistletoe preparations” depending on the setting of the parameters.

Figure 3 illustrates the behaviour of the search algorithm for the threshold values *Q_t_* = 35 and *Q_t_* = 45 and a high (+) versus a low (−) sensitivity. Note that the threshold values *Q_t_* = 35 together with a low sensitivity results in a total of 54 hits whereas *Q_t_* = 45 with a high sensitivity delivers only 10 hits. The remaining two combinations *Q_t_* = 45 with low sensitivity and *Q_t_* = 35 with a high sensitivity each achieve about 20 hits. Also note that in these cases the decrease in quality of the hits develops slightly differently.

## Inverted File Structure

**Figure 4:**
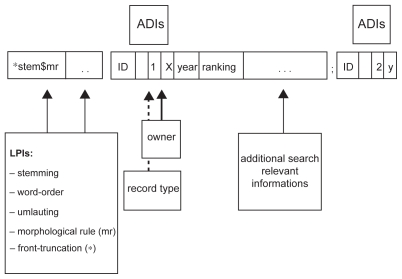
Schematic description of an inverted file structure of a bibliographical dataset with linguistic processing information in the front and additional discriminant information (ADIs) in the back of the coding. “*stem$mr” denotes the decomposition of a search term i.e. “oncology” leads to “oncolog$~y”. Note that front truncation information (marked with a “*”) and umlauting are features specially designed for German language (i.e. for composition terms like “Krebstherapie” (engl. “therapy of cancer”).

In our context, along with the linguistic processing of the bibliographical metadata of documents, data volume and query processing loads increase continually and thus it is of great importance to have an efficient information retrieval system which is able to process the search queries in an appropriate run time. We therefore decided to use an indexing mechanism based on inverted file structure.^22^ All bibliographical datasets (including sentences if an abstract is available) are processed into an index by the above mentioned linguistic algorithms, and together with additional discriminating information (ADIs) for example, the logical subset that a bibliographical record belongs to, an inverted file structure is created (Fig. 4).

Within the context of linguistic indexing, one new feature has been developed to enhance the speed of search algorithms operating on conventional inverted file structures: words are no longer regarded as textual ‘atoms’, but are recognized in their syntactic-semantic interrelation. Based on a 6-byte coding, the above mentioned mathematical algorithms produce a ‘word-map’ of the documents which provides the information on whether or not a word appears in certain documents. This information is used as a filter when more than two search terms are entered in the query. Thus, matching-algorithms may abstain from processing a document from the outset if the word-map provides this information, which increases the speed of processing the search query.

### Example 2: Search-query ‘mistletoe in oncology’

When searching for the phrase “adverse effects of mistletoe preparations”, our matching algorithms, based on the mathematical calculation of Q (adverse, effects, mistletoe, preparations), together with the inverse file coding, are able to detect whether the term “preparation~s” occurs in a dataset or not. Thus, documents found in the search for ‘mistletoe’ without the words “preparation~s” or “adverse effect~s” are not processed any further because no match for the combined search term emerges. This has quite a dramatic effect on the processing speed of the search query. In the search query example given above, the single term “adverse” results in a performance of 0.05 seconds based on the average of 20 subsequent entries on a conventional notebook with a DSL-6Mbit data rate. For the terms “adverse” and “effects”, the database responds with a mean of 0.06 seconds. Adding the search term “mistletoe” increases the speed by a factor of 6 to 0.01 seconds and finally the complete phrase is processed in 0.005 seconds on average.

**Figure 5:**
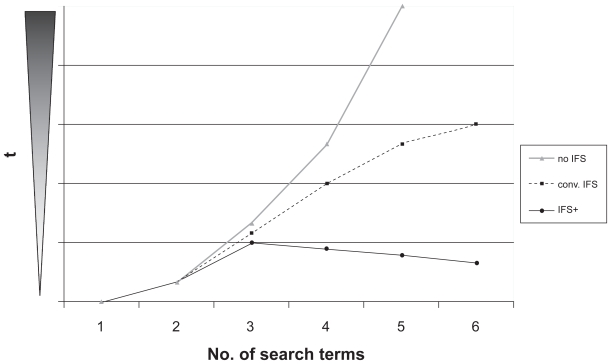
Illustration of seeking time *t* behaviour in relation to the number of search terms for search queries processed by conventional search algorithms without IFS (no IFS), conventional search algorithms operating on inverted file structures (IFS) and special algorithms given in CAMbase operating on inverted file structures (IFS+). Whilst conventional structures without IFS increase exponentially in their seeking time, IFS has a more or less logistic curve, and IFS+ decreases in its searching time the more search terms are included in the query.

As shown in the example, our method is more efficient when more search terms are entered in the search query, in contrast to conventional data structures which at first sight shows a paradoxical search time behaviour as illustrated in Figure 5. This procedure becomes more important with larger data volumes and is also used for keyword landscapes described below.

## Semantic Web Standard: XML

As bibliographical data, in the case of CAM, does contain several heterogeneous elements arising from the different sources from which the datasets originate (i.e. publishers, libraries, authors entries, offline collections of datasets of research institutes), we decided to use the innovative technological web-standard XML (eXtended Markup Language)^23^ to receive capture the output in a homogeneous structure. We constructed XML-based import interfaces to process incoming documents of varying document type definitions (DTDs), to extract structural and descriptive metadata from these documents and to deliver special document output styles on demand. As an additional feature, we also implemented XML-interfaces for the standards given by the Open— Archives—Initiative (OAI). With this XML—based document-management, CAMbase can be easily connected with national and international electronic databases and other digital libraries.^24^,^25^ In addition, connections with other metadata, e.g. semantic web standard ontologies, can be established.

The basic idea behind this approach is to guide the user through the bibliographical landscape. The following figure illustrates this approach with the example of the search query for “Krebs” (German for “cancer”).

Given the German language search term “Krebs”, the search algorithm which is similar to other conventional databases displays a listing of articles which includes this search term or compositions like “Krebstherapie” (Engl: “cancer therapy”). Additionally, a keyword-landscape on the right side (German Title: “Themenlandkarte”) is also given which describes the resulting dataset more precisely. Keywords occurring more often are displayed in the landscape with a bigger font size and at least one dataset behind every keyword displayed in the landscape is guaranteed.

**Figure 6:**
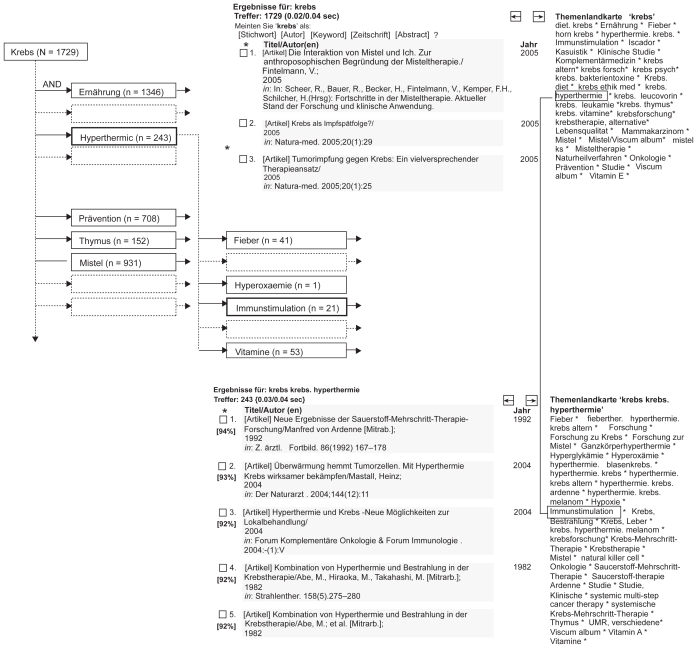
Search tree and respective screen shots of CAM-landscapes on the search query “Krebs” (engl.: cancer).

By clicking on a term displayed in the landscape, the system again displays the results. In our case, clicking on the term “Hyperthermie” leads to 243 hits which are equivalent to a boolean AND-relation “Krebs AND Hyperthermie”). Naturally, the results of this search query are also linked to another landscape (see Fig. 6). With each navigation step, the user might choose another keyword for narrowing the result set even further. An additional feature is available after the nth step (n > 2) offering the user permutations of the search terms of the (n−1)th step: For the query “Krebs AND Hyperthermie AND Immunstimulation” (2 hits), the system also asks whether the user might want to switch to the search query “Krebs AND Immunstimulation” without the term “Hyperthermie” (19 hits).

## Discussion

The next generation of the Web is often characterized as the “Semantic Web”^26^ in which information processing is no longer the task for human users, but rather handled by machines and algorithms giving rise to semantically empowered search engines such as the one described above. Semantic processing, however, requires standards for the syntactic form and for the semantic content of documents. One important technology for this purpose is already in place: eXtensible Markup Language (XML).

There are other semantic web standards used for indexing bibliographic resources, like Web Ontology Language (OWL) and Resource Description Framework (RDF), that can facilitate data integration i.e. for genomic data^27^ and applications that employ ontologies such as indexes and query models, e.g. GoPubMed,^28^ Knowlegator^29^ and Textpresso).^30^,^31^ These systems show sophisticated use of of bibliographic metadata for indexing literature abstracts or full texts according to domain content. All of these approaches are domain specific and the approach presented here is likewise a first for the field of CAM.

A first evaluation of the vocabulary used in more than 28,000 search queries applied to CAMbase between 2003 and 2006 found that 12.2% were regarding authors publishing primarily in CAM, 10.9% on general terms, 30.3% on diseases, disorders and symptoms, 25.2% on therapies and procedures and 10.2% on plants and ingredients.^32^ However, according to the recently conducted review of Kim et al^33^ of tag ontologies for semantically linked data, a reorientation in evaluation strategies seems to be necessary to develop appropriate metrics, particularly if the results of this evaluation shall be used for the creation of a CAM-ontology.

In particular, a precise identification of entities is essential for the creation of an ontology-driven document indexing and data integration.^34^ In addition, most of our data can be considered to be “old data”, which means, there is only limited information for the creation of a valid ontology and changes in underlying ontologies might have far-reaching side effects for more recent data as the old scheme might not be compatible with the new results.^35^ Nevertheless, if one looks closely at the CAM-landscapes created for every search query they implicitly indicate the presence of some underlying ontology. Thus, according to Vatant^36^ a closer analysis of our CAM landscapes would be a first step to establish an ontological framework of CAM. Within this context the use of RDFs as interchange formats and the OWL as a formal description of the underlying concepts, terms, and relationships seem promising.

Taking into consideration the high amount of German language articles on CAM, our features and algorithms even today are of direct relevance. Whilst we have addressed the technical specifications in the construction of CAMbase, the issue of generating semantic ontologies in CAM is even more challenging than in other disciplines. This is because a catalogue of homogeneous synonyms for medical terms (which is valid for all CAM-disciplines) remains intangible (i.e. the term “liver” has a quite different meaning in traditional Chinese medicine than in homeopathy).

Mertz^37^ also pointed out that one of the important future tasks for research in CAM is to build CAM vocabularies and make them available to the public domain. In doing so, one has to be aware that even if some values are shared with traditional biomedicine, axiological differences have to be considered as well. Although our examples are based on CAM bibliographical records in “CAMbase”, the underlying algorithms we have developed are generic and work for other contexts as well. For example, a user might not only want to access the scientific literature, but also wants to look for a research institution or an expert involved in a distinct search topic (i.e. for a unique cancer treatment). In the same way as described above, this can be realized with an XML-based content management system, in which such information is stored^24^ and a multi-dimensional web-portal is the next logical step.

Another example where deployments of semantic ontologies can be found within the field of CAM is the use of these features and tools for electronic lexicography.^38^,^39^ In particular, machine readable lexicons or dictionaries with a codification of domain knowledge and literature metadata in accordance with a generic and extendible XML scheme model are more suitable in this context. One open problem in complementary medicine in this context is the semantic processing of the many resources of homeopathic remedies, *Materia Medicia*. Such repositories contain structured data on medical symptoms and the corresponding homeopathic remedies. Natural language processing of and ontology creation for such vast resources would be beneficial in the complex process of homeopathic prescribing.^40^

## Conclusion

Although the internet has brought a ‘revolution to information technology’, most of the current forms of web content remain incomprehensible for intelligent analysis by computers. The semantic web standard XML can be used to unite structural properties of databases, web requirements and the demands of end users. In this publication we were able to show how XML together with other algorithmic features like linguistic processing may aid the user to find his/her way through incomplete and often only semi-structured data in the field of CAM. On a long term, our approach may lead to a creation of a CAM ontology as currently described for other areas of biomedical research.^41^

## Figures and Tables

**Table 1:** Description and Examples of NLP-techniques applied in CAMbase.

NLP-Technique	Description	Examples
**Morphological analysis of**		
• Flexions	Change of word form which does not change the part-of-speech category, such as conjugation	Treat/-ing/-ed; assess/-ment
• Compound words	Rule based decomposition of a word into its base forms; base form compounding	Krebstherapie (engl.: cancer therapy) is decomposed into “Krebs” and “Therapie”; “Lektine” (engl.: lectins) also finds “Mistellektine” (engl.: “mistletoe lectins”)
• Irregular plurals	Rule based analysis of German	“Fragebogen” (engl.: questionnaire) finds “Fragebögen” and vice versa
**Stemming and Lemmatisation**		
• Stemming	Rule based stripping off affixes to get the stem (root) of the word	German: Chemotherap/-ie/-n; English: filter/-s
**Grammatical analysis**		
• Subject-Object relations	Grammatical dissolution and analysis of the search phrase	See Figure 2
**Tokenisation/Text Segmentation**		
• Punctuation	Splitting the character sequence at white space positions.	
• Normalisation	Analysis of capitalised words.	MIT (Massachusetts Institute of Technology) is different from the German “mit” (engl: “with”)
**Word Distributions**		
• Analysing word frequencies and collocations	Comparing different uses and occurrences of the same word/stem	Thematic landscapes; Tag clouds (see Fig. 6)
